# Hospital Meetings, &C.

**Published:** 1898-07-09

**Authors:** 


### HOSPITAL MEETINGS, &C.

### HOSPITAL FOR DISEASES OF THE THROAT.

On Wednesday, 29th ult., the annual general meeting of the Hospital for Diseases of the Throat, Golden Square, was held in the Medical Society's Rooms, Chandos Street, Cavendish Square, Mr. H. G. Pember, the chairman of the Committee of Management, presiding. In moving the adoption of the report and accounts, Mr. Pember referred to the reason for his occupancy of the chair—the lamented death of the late president, Earl Strafford, and a vote was passed expressing the meeting's sense of sorrow at the loss of so worthy a president. No decision has yet been come to about filling the vacant post, which is expected to be a task of some difficulty. The past year, both for the Committee of Management and for the medical staff, was one of exceptional strain, owing to the disturbed state of the hospital due to the building operations which have been going on, but which are now happily almost complete. The extension will give greatly increased accommodation for out-patients and staff, as well as 25 extra beds for in-patients, just double the number formerly available. But, as the Chairman was careful to point out, the increased building and establishment mean increased outlay, and necessitate increased exertions on the part of all friends of the institution to provide the extra funds required. The amount of the "Morell Mackenzie Memorial Fund" (£1,023) has been applied towards the extension. Owing to the interference with the work of the hospital caused by the building operations, the number of patients shows a slight falling off. As compared with 519 in 1896, the in-patients were 470. In 1896 the out-patients were 8,881; last year 8,581. The attendances in 1897 numbered 41,602, against 43,272 in 1896. The ordinary income for the year was £2,698, plus legacies £650, and the ordinary expenditure amounted to £3,841. It is proposed to make considerable additions to the medical staff.

